# Expression, processing and transcriptional regulation of granulysin in short-term activated human lymphocytes

**DOI:** 10.1186/1471-2172-8-9

**Published:** 2007-06-27

**Authors:** Sonja Latinovic-Golic, Michael Walch, Hanna Sundstrom, Claudia Dumrese, Peter Groscurth, Urs Ziegler

**Affiliations:** 1 Division of Cell Biology, Institute of Anatomy, Winterthurerstr.190, 8057 Zürich, Switzerland

## Abstract

**Background:**

Granulysin, a cytotoxic protein expressed in human natural killer cells and activated T lymphocytes, exhibits cytolytic activity against a variety of intracellular microbes. Expression and transcription have been partially characterised in vitro and four transcripts (NKG5, 519, 520, and 522) were identified. However, only a single protein product of 15 kDa was found, which is subsequently processed to an active 9 kDa protein.

**Results:**

In this study we investigated generation of granulysin in lymphokine activated killer (LAK) cells and antigen (*Listeria*) specific T-cells. Semiquantitative RT-PCR revealed NKG5 to be the most prominent transcript. It was found to be up-regulated in a time-dependent manner in LAK cells and antigen specific T-cells and their subsets. Two isoforms of 519 mRNA were up-regulated under IL-2 and antigen stimulation. Moreover, two novel transcripts, without any known function, comprising solely parts of the 5 prime region of the primary transcript, were detected. A significant increase of granulysin expressing LAK cells as well as antigen specific T-cells was shown by fluorescence microscopy. On the subset level, increase in CD4^+ ^granulysin expressing cells was found only under antigen stimulation.

Immunoblotting showed the 15 kDa form of granulysin to be present in the first week of stimulation either with IL-2 or with bacterial antigen. Substantial processing to the 9 kDa form was detected during the first week in LAK cells and in the second week in antigen specific T-cells.

**Conclusion:**

This first comprehensive study of granulysin gene regulation in primary cultured human lymphocytes shows that the regulation of granulysin synthesis in response to IL-2 or bacterial antigen stimulation occurs at several levels: RNA expression, extensive alternative splicing and posttranslational processing.

## Background

A crucial role in destroying tumor cells, virus infected cells and intracellular pathogens is played by natural killer cells (NK) cells and cytotoxic T lymphocytes (CTL) [1-3]. They are armed with cytotoxic granules containing various effector molecules such as granulysin, perforin, and several granzymes [4,5]. Granulysin, an antimicrobial protein, was first identified by a subtractive hybridization procedure in functional T cell lines and alloantigen and mitogen stimulated PBL and was initially referred to as 519 [6]. A similar gene product, referred to as NKG5, was found in the NK cell line EDF as well as in different T cell lines and clones [7]. Furthermore, Manning et al. [8] investigated the structure and transcription of the granulysin gene in the functional T cell line AJY and found the alternative spliced transcripts 519, 520, and 522 but no NKG5.

The granulysin gene is located on the human chromosome 2 [9] and comprises 6 exons within a 3.9 kb genomic locus encoding at least four alternatively spliced transcripts (NKG5, 519, 520 and 522), all differing mostly in exon 2. The most abundant transcript in AJY was identified to be 520 appearing late after T cell activation [8]. Since 520 has an appropriate Kozak sequence for the initiation of translation, this transcript was thought to be responsible for the majority of granulysin protein produced [5]. NKG5, which is very similar to 520, but is absent in the AJY, would have the same function in NK cells.

Granulysin is expressed as a 15 kDa protein which is processed by proteolytic cleavage at both N and C termini of the 15 kDa precursor to the active 9 kDa form [10]. Whereas the 15 kDa protein is produced rapidly, has a shorter half-life and is poorly secreted, the secretory 9 kDa form is produced slowly and is relatively stable [5]. Recombinant 9 kDa granulysin exhibits cytolytic activity against microbial and tumor targets [3,11,12].

Overall, the complex transcriptional activity of the granulysin gene was found by studying different cell lines but a comprehensive analysis of the granulysin expression in primary cultured lymphocytes has not been performed. It has been shown that granulysin expression is induced in different lymphocyte subsets in response to bacterial antigens [13-15].

In this study, we examined the expression and processing of granulysin in non-transformed lymphocytes which were either stimulated with r-IL-2 (lymphokine activated killer cells LAK cells) or primed by autologous dendritic cells (DC). We used two different bacterial antigens, *Listeria innocua *and *Listeria monocytogenes*, which are presented to the immune system in different ways While *L. innocua *is Gram-positive, non pathogenic, ubiquitous bacterium, which may be processed and presented via the MHC class II pathway, *L. monocytogenes *is an intracellular pathogen, which can enter the cytosol of infected host cell allowing processing for presentation on MHC class I molecules [16-18]. Both Listeria species have been found to be successfully killed by granulysin [11,12].

## Results

### Transcriptional regulation of granulysin in LAK cells

Transcriptional analysis of the granulysin expression has been assessed in cell lines but not in primary cultured lymphocytes. To address this issue we investigated the transcripts encoded by the granulysin gene in freshly isolated PBMC stimulated with IL-2 for 2 weeks.

**Figure 1:**
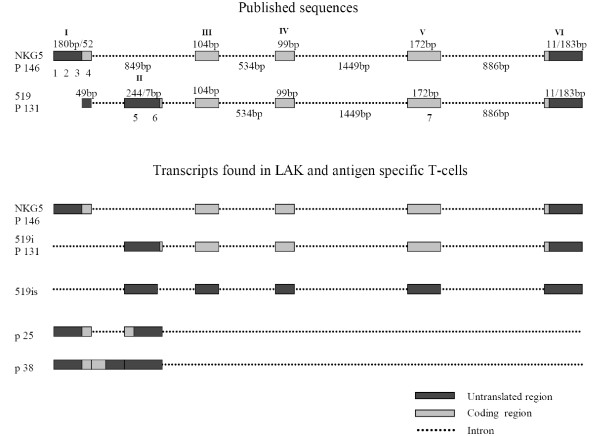
**Schematic view of known and found granulysin transcripts**. Upper part of the figure shows schematically transcripts from GenBank and down part transcripts that were found in LAK or antigen specific T-cells. Black boxes indicate coding and gray boxes noncoding exon parts. Exons are marked with roman numbers. Dotted lines correspond to intron regions. Arabic numbers 1 to 7 stay for primers numbered in the same way as in Table 1.

**Figure 2:**
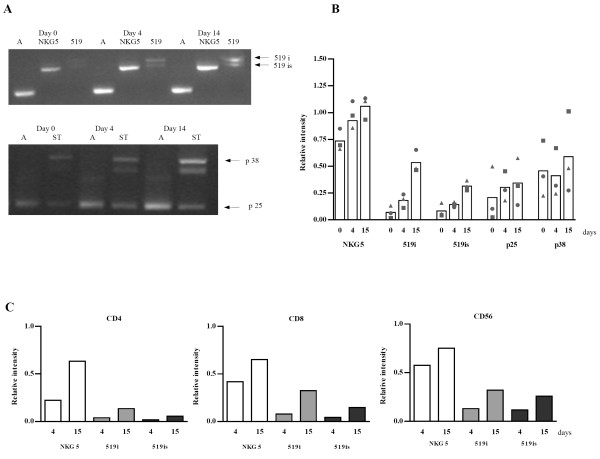
**Granulysin gene expression in LAK cells and different subpopulations**. LAK cells were cultivated within 2 weeks under continuous IL-2 stimulation. At indicated time points cell samples were taken for RT-PCR analysis. Representative gel image shows transcripts present in LAK cells over the stimulation period (A). The same transcripts were found in different subpopulations (not shown). Graph (B) represents results of semiquantitative analysis of the transcripts normalized to actin (gel image, lines A) in LAK cells from three different donors. Each symbol represents one donor and bars correspond to the mean value. Graph (C) shows the same analysis in different subpopulations where one representative donor is shown.

LAK cells showed high expression of NKG5, of two isoforms of 519 mRNA and two novel transcripts (Fig. 2A). Regarding the transcripts published by Manning et al. [8] two sense primers in exon 1 (primer 2 and 3 (Fig. 1)) were tested but failed to expand the mRNAs corresponding to 520 or 522 (data not shown). However, all tested sense primers in exon 1 (primer 1, 2, 3, 4 (Fig. 1)) and a reverse primer in exon 5 (primer 7) resulted in a single PCR product (Fig. 2A) that was identified by sequencing as the published transcript variant NKG5 (GenBank NM 006433). Both products obtained with the 519 primer (primer 5 (Fig. 1)) were also sequenced and identified to be similar to the 519 transcript published by Manning et al. [8]. Both isoforms differ from the published transcript of 519 by not containing any part of exon 1 (transcripts are called 519i throughout this paper) and one isoform additionally lacks 81 nucleotides at the 3' end of exon 2 (519is, see Fig. 1).

To investigate whether any transcript includes exons 1 and 2, we performed a RT-PCR using the NKG5 sense B (primer 2) and an antisense primer in exon 2 (primer 6). By this approach two novel short transcripts (ST) were found in LAK cells and in all subsets (Fig. 2A, arrows p38, p25). Sequence analysis revealed ST p25 to comprise exon 1 and exon 2 (see Fig. 1) and ST p38, comprised of p25 plus the whole intron 1, as published by Manning et al. [8]. None of the ST extended to exon 3 (data not shown). Since both ST contain a Kozak consensus translation initiation site, they could be translated into peptides with a length of 25 and 38 amino acids, respectively. The middle band that appears on the agarose gel (Fig. 2A) is the hybridization product of the two ST, with loop formation within intron 1. This was proven by 15 repeated denaturation and annealing steps of the extracted middle band resulting again in 3 bands (data not shown).

Semi-quantitative analysis of the PCR products revealed that in the total population of LAK cells, the NKG5 message increased only slightly by a factor of 1.5 from day 0 to day 15 (p = 0.019), while the two variants of 519 increased by a factor 8.8 (519i; p = 0.002) and 4.4 (519is; p = 0.007), respectively (Fig. 2B).

The ST did not show an apparent IL-2 dependency. Since each donor exhibited a different regulation pattern, a clear tendency for up- or down regulation in response to IL-2 could not be determined (Fig. 2B).

Next, we examined the expression of the various longer transcripts in T cell subsets. Using magnetic beads, CD4^+^, CD8^+ ^and CD56^+ ^cells were isolated at days 4 and 15 of continuous IL-2 stimulation and RT-PCR analysis was performed. As shown in Figure 2C the highest expressed transcript in all subsets at day 4 is NKG5, while the 519 isoforms are poorly expressed, especially in CD4^+ ^cells. During continuous IL-2 stimulation all transcripts analyzed were at least slightly upregulated in the different cell subsets in all donors tested (Fig. 2C, one representative donor shown).

Short transcripts were also detected in all subsets at days 4 and 15, but their kinetics of expression were not evaluated (data not shown).

### Transcriptional regulation of granulysin in antigen specific T-cells

**Figure 3:**
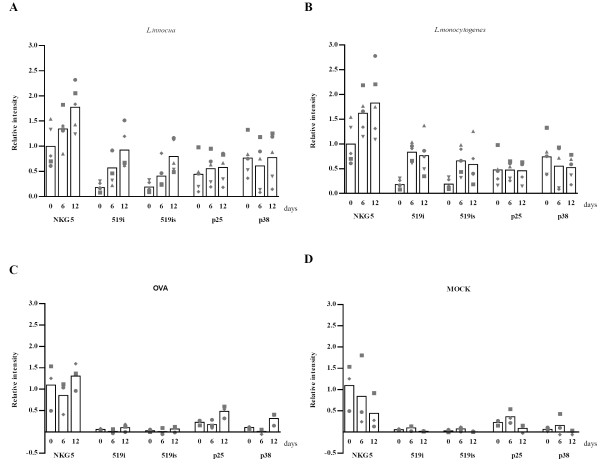
**Granulysin gene expression in antigen specific T-cells**. *L. innocua *(A) respectively *L. monocytogenes *(B) OVA (C) and MOCK (D) specific T-cells were cultivated as described in Materials and methods. Samples were collected before stimulation (day 0), before restimulation (day 6) and 6 days after restimulation (day 12). PCR was performed and analysed by gel electrophoresis. Values are results from semiquantitative analysis of transcripts normalised to actin from the same cDNA. Each symbol represents one donor.

Host defense mechanisms against intracellular microbes require induction of effector molecules such as granulysin during infection. Therefore, we investigated if granulysin gene expression is induced upon antigen driven stimulation of T cells and if so which of the different granulysin transcripts might be involved. *L. innocua *and *L. monocytogenes *were used as antigens in mixed leukocyte culture. The expression of granulysin transcripts was investigated in antigen specific T-cells from autologous MLCs by RT-PCR. The identical mRNAs found in LAK cells were detected in the T-cells stimulated with *L. innocua *or *L. monocytogenes*, including the ST (data not shown). Semi-quantitative analysis of the transcripts (Fig. 3) revealed that NKG5 mRNA showed the highest basal expression at day 0 and was upregulated between days 0 and 12 by an average factor of 1.7 (*L. innocua*, x1.68, p = 0.02; *L. monocytogenes*, x1.7, p = 0.002). Both 519 isoforms showed a more prominent increase between day 0 and day 12 of stimulation by an average factor of 3.8 (519i) and 2.7 (519is), respectively (*L. innocua*, x4 519i, p = 0.004 and x2.7 519is p = 0.006; *L monocytogenes*, x3.6 519i, p = 0.0004 and x2.8 for 519is p = 0.01). In *L. innocua *specific T cells 519 isoforms were continuously increasing during the whole period of antigen stimulation in contrast to *L. monocytogenes *specific T cells where the increase was rapid in the first weak of stimulation but remained unchanged during the second weak. Semi-quantitative analysis of short transcripts did not allow us to see any tendency for up- or down-regulation under bacterial stimulation. OVA specific T cells maintained basal expression levels of NKG5 mRNA during the stimulation period, with no upregulation. The two 519 isoforms remained at very low levels of expression over the whole period of stimulation. Short transcripts showed a slight increase, which was statistically significant (p25, p = 0.039; p38, P = 0.029). None of the transcripts were upregulated in T cells where MOCK treated DCs were used in MLC.

In T cell subsets all transcripts, but especially the 519 isoforms showed prominent upregulation between day 0 and 6. Between day 6 and day 12 no significant difference in the mRNA levels was found (data not shown). Overall these data indicate that especially the two 519 isoforms seem to be tightly regulated on transcriptional level in response to either antigen or IL-2 stimulation

### Granulysin expression in LAK cells

**Figure 4:**
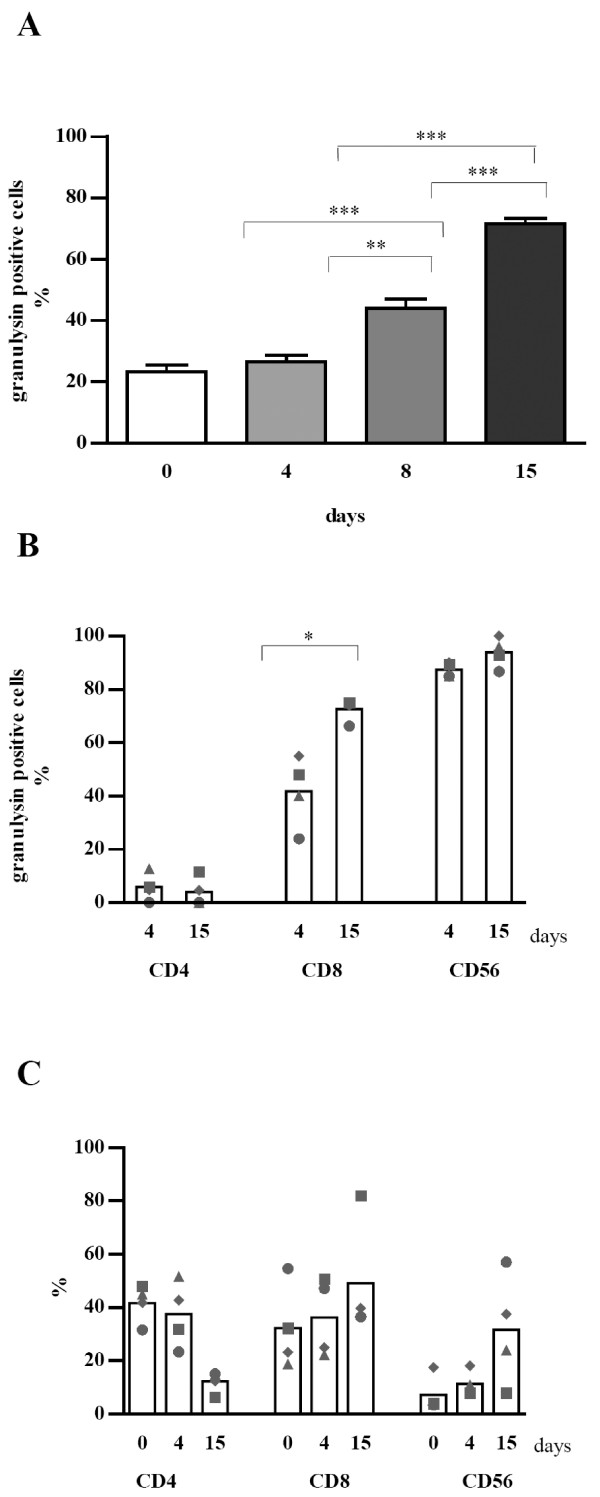
**Time dependent expression of granulysin in LAK cells and different subpopulations. **LAK cells (A) or different subpopulations (B) were stained for granulysin and CD surface markers. Cells were analysed using confocal laser scanning microscope by counting 500 cells pro sample. Graph (C) represents phenotype analysis of LAK cells. Four different donors were analysed and each symbol represents one donor. Bars indicate mean values. (* p < 0.05; * * p < 0.005; * * * p < 0.0005)

To follow the granulysin expression under continuous IL-2 stimulation, LAK cells were stained for granulysin at indicated time points and analyzed by counting granulysin positive cells. The expression of granulysin within the whole LAK population was not changed after 4 days but a significant increase became apparent after 8 and 15 days of IL-2 stimulation (Fig. 4A). Granulysin expression in different subsets of LAK cells was investigated by double labeling for CD surface markers and granulysin followed by counting of at least 500 cells. Significant increase in granulysin expression could only been seen in CD8^+ ^cells from day 4 to day 15, while CD56^+ ^cells expressed granulysin already at day 4 at very high level and no significant alteration was observed until day 15 (Fig. 4B). Only a few CD4^+ ^cells were found to be positive for granulysin over the whole period of stimulation. The phenotypic analysis of LAK cells revealed that especially the number of CD56^+ ^and the CD8^+ ^cells increased, while the CD4^+ ^cells decreased over time (Fig. 4C). The phenotypic pattern of LAK cells under IL-2 stimulation varied between the different donors. This is consistent with former results obtained by phenotyping PBMC of different donors under IL-2 stimulation [19]. But as a trend in most of the donors the number of CD56^+ ^and CD8^+ ^cells was rising while the number of CD4^+ ^cells was decreasing over time.

These data indicate that in LAK cells the increase in granulysin expression is mostly due to the increasing number and expression level in CD8^+ ^cells as well as the increasing number of CD56^+ ^cells during IL-2 stimulation.

**Figure 5:**
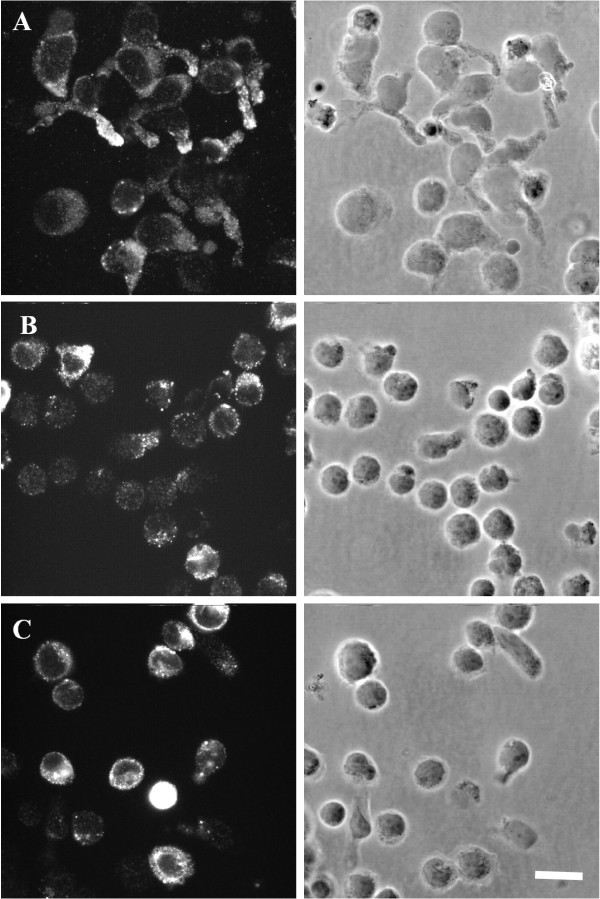
**Confocal microscopy of LAK and Listeria specific T cells labeled for granulysin**. LAK cells (A) after 15 days of IL-2 stimulation, *L. innocua *(B) as well as *L. monocytogenes *specific T cells (C) after 12 days culture. Bar 10 μm.

The spatial pattern of the granulysin distribution in LAK cells was examined by immunostaining for granulysin using a polyclonal anti-granulysin Ab, which allows the detection of the 15 kDa and 9 kDa form of granulysin. Confocal microscopy analysis at day 15 of stimulation revealed a vesicular staining pattern in highly polarized cells consistent with the granular localization of granulysin (Fig. 5A).

### Expression of granulysin in antigen specific T-cells

**Figure 6:**
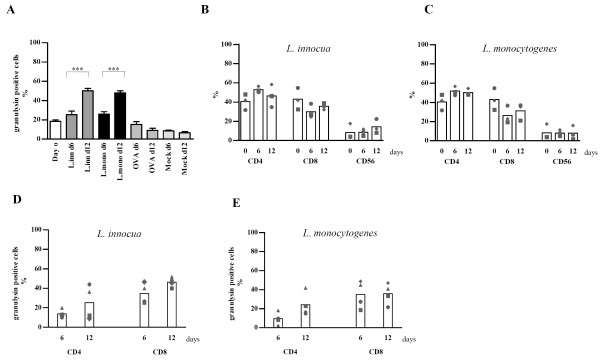
**Expression of granulysin over time in antigen specific T cells and different subsets**. T-cells were removed from mixed lymphocyte culture at selected time points and stained with anti-granulysin Ab. Cells were analysed with a confocal laser scanning microscope counting 300 cells. Graph (A) shows granulysin expression in antigen specific T-cells as a whole population; (B) represent phenotype of *L. innocua *specific T cells; (C) phenotype of L. *monocytogenes *specific T cells graph (D) represent granulysin expression in different subsets in *L. innocua *specific T cells and graph (E) in *L. monocytogenes *specific T cells. In (B), (C), (D), and (E) every donor is indicated with one symbol. Bars are mean values. (*** p < 0.0005)

To determine the regulation of granulysin expression upon bacterial antigen stimulation, antigen specific T cells were stained with anti-granulysin Ab at days 0, 6 and 12. The percentage of granulysin positive cells was determined by counting at least 300 cells. Granulysin expression was induced after 6 and 12 days from 18% to around 50% granulysin positive cells in response to both bacterial antigens. Cells stimulated with OVA or MOCK did not show any increase in granulysin expression over time (Fig. 6A).

The T cell population consisted mostly of CD4^+ ^and CD8^+ ^cells with only moderate changes over time as assessed by flow cytometry (Fig. 6B and 6C).

Analysis of granulysin expression in different subpopulations was done in CD4^+ ^and CD8^+ ^cells separated at day 6 and day 12 and stained for granulysin. In contrast to IL-2 stimulated LAK cells, in *L. innocua *as well as in *L. monocytogenes *specific T cells, around 30% CD4^+ ^cells expressed granulysin. Approximately 40% of the CD8^+ ^cells expressed granulysin at day 12 under these conditions (Fig. 6D and 6E). There was a very similar pattern of expression in different subpopulations in the differently primed T cells allowing no conclusion on the preferential presentation in MHC I and MHC II, respectively. As apparent in the graphs there was a higher scattering in the granulysin expression in subsets of cells after antigen specific stimulation compared to LAK cells. This reflects the observation that the T cells of some of the donors were highly responsive to *Listeria *antigens while others showed only a slight increase in granulysin expression.

High resolution imaging of *Listeria-*specific T cells after granulysin immunostaining revealed a granular and slightly polarized pattern of granulysin in round shaped cells that are smaller in size compared to LAK cells (Fig. 5B and 5C).

### Processing of granulysin in LAK cells and antigen specific T-cells

**Figure 7:**
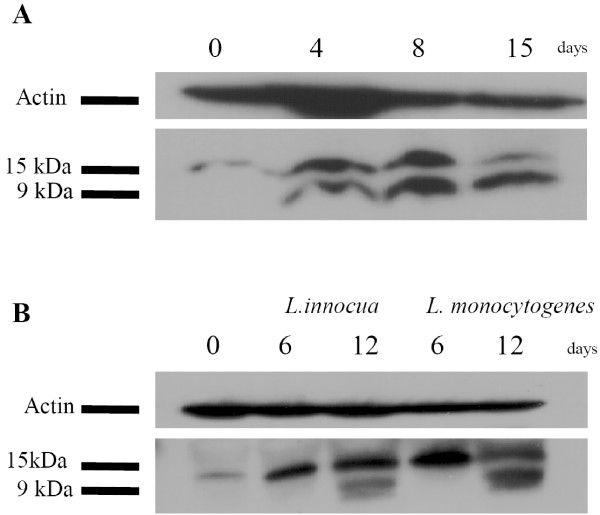
**Kinetic of granulysin processing in LAK and Listeria specific T cells**. LAK (A) or antigen specific T cells (B) were taken out of culture at indicated time points followed by lysis of cells and separation of proteins by SDS-PAGE. Proteins were blotted and detected using anti-granulysin Ab (1:1000). The blot was stripped and probed for the β-actin.

Pena et al. [3] showed granulysin to be expressed as 15 kDa and 9 kDa forms in CTL cell lines, late after activation with alloantigen. To investigate which protein forms are induced upon IL-2 stimulation, LAK cells were analyzed at indicated time points using Western blot analysis. In unstimulated cells only the 15 kDa protein was detectable. During the IL-2 stimulation the 15 kDa precursor became more prominent while the active 9 kDa form was first visible after 4 days of stimulation. In the second week of stimulation the signal of the 15 kDa protein diminished and a strong 9 kDa band was detected (Fig. 7A). This was compared to the kinetics of protein processing under antigen stimulation. As shown in Figure 7B*Listeria *specific T-cells accumulated the 15 kDa precursor during the whole period of stimulation while the 9 kDa protein was first detected in the second week of stimulation.

## Discussion

Activation of T-cells and NK cells can occur through a wide range of stimuli, including bacterial, tumour and viral antigens or cytokines. As a result, these cells express a number of different cytotoxic proteins such as perforin or granzymes to kill target cells or granulysin for killing intracellular bacteria [6,20-23]. The expression and regulation of granulysin has so far only been studied in cell lines of NK and T-cell origin [8], where the transcripts NKG5, 520, 522 and 519 have been found. To a limited extent, 519 expression in response to mitogen and alloantigen stimulation was also studied in PBMC [6]. NKG5 was found only in a NK cell line [8] and was therefore thought to be a NK specific transcript. Transcripts 519, 520 and 522 had been found in AJY, a functional T-cell line [8]. Our newly described variants of 519 as well as the novel short transcripts were not found to be expressed in AJY. On the other side, we could not detect 522 and 520 mRNAs described in AJY. In our system all transcripts detected were expressed both in NK and T-cells. Another striking difference between transcript 519, found in the cell line AJY, and the 519 isoforms in PBMC derived LAK and *Listeria *specific T-cells, is the absence of exon 1. Currently, it can only be speculated if all these differences are due to a different regulation of granulysin gene expression in primary cultured cells. Of longer transcripts, the constitutively expressed NKG5 mRNA has an appropriate Kozak consensus sequence and a putative hydrophobic leader cleavage site and, as we found, it is also expressed in T-cells. It is most likely the transcript encoding the 15 kDa protein form that is then processed to the active 9 kDa secreted protein in NK and T-cells. In contrast to the NKG5 transcript, the two 519 isoform mRNAs had a very low basal expression and were significantly and strictly upregulated by IL-2 and bacterial antigen, indicating possible contribution of these alternative spliced transcripts in the transcriptional regulation of the granulysin gene. The longer form of the 519 isoform has also one potential in-frame initiation codon but it lacks the Kozak consensus as well as a hydrophobic leader sequence which would suggest that little or no secreted protein is encoded by this transcript. It has indeed been shown that transient expression of 519 cDNA in COS-7 cells did not produce a detectable level of protein [5]. As the 519 short variant does not have an in-frame start codon, its possible impact is rather unclear. The short transcripts (p25 and p38) have the potential to be translated since they could use the same start codon as NKG5 and could enter the secretory pathway, but no experimental evidence is yet available.

As experimental evidence is still missing, we can only speculate how the transcriptional regulation of granulysin gene expression is mediated. The granulysin gene shows extensive alternative splicing which is in contrast to other cytotoxic proteins, such as perforin and granzyme B encoded by only one transcript [6,24,25]. For the vast majority of alternative splicing events, the functional significance is unknown. The potential role of alternative splicing and different protein isoforms in the regulation of transcriptional activity has already been reported in humans e.g. for p53 and GLI2 proteins [26,27]. Willingham et al. found several functional non coding RNAs. Some of them are essential for cell viability, one repressor of Hedgehog signaling and one that acts as repressor of transcription factor NFAT, which is itself required for T-cell receptor-mediated immune response [28]. Some possible functions of non-coding transcripts could be stabilizing of coding transcript or post-transcriptional regulation of gene expression [29,30]. To find out whether the two novel transcripts (p38 and p25) are translated in CTLs, their possible roles and the function of the 519 isoforms needs further investigations.

It has been reported that primary human NK cells constitutively express granulysin whereas CD8^+ ^cells express granulysin upon stimulation [31-33]. Our results revealed a significant increase in protein levels over time under IL-2 or antigen stimulation observed in the whole cell population Monitoring different subpopulations, IL-2 increased protein synthesis only in CD8^+^cells. Recently Zheng et al. [34] showed that IL-2 induces granulysin expression in isolated CD4^+ ^cells. In our system, IL-2 did not induce upregulation of granulysin expression in CD4^+ ^cells, but all transcripts were induced in a time dependent manner. Approximately 10–15% CD4^+ ^cells expressed granulysin between the first and second week of IL-2 stimulation. This discrepancy could be due to the fact that we did not isolate CD4^+ ^out of the whole population prior to stimulation. Similar to our results, Liu et al. showed that IL-2 induced expression of perforin and granzyme A and B was mostly seen in CD8^+ ^subset, but in CD4^+ ^cells they also detected only mRNA [20,35]. As expected, the CD56^+ ^population showed very high constitutive expression of granulysin protein and messenger. When stimulated with IL-2, CD56^+ ^cells proliferate and after 2 weeks constitute around a half of the whole LAK cell population [19]. Taken together, increased levels of granulysin under IL-2 stimulation, monitored in the whole LAK population is due to an increasing number of CD56^+ ^and CD8^+ ^cells as well as due to the induction of the protein synthesis in CD8^+ ^cells. The number of CD4^+ ^cells decreased under IL-2 stimulation, which could be explained by different expression of IL-2 receptor chains in different T cell subsets [20,36,37].

A slightly different pattern was observed in T cells specific for *L. innocua *or *L. monocytogenes*, where stimulation with both bacterial antigens revealed a similar phenotype with almost equal proliferation of CD4^+ ^and CD8^+ ^cells. This is in agreement with other studies showing that *Listeria *antigens may be presented via MHC class I and class II molecules [38-40]. Similar to IL-2 stimulation, the increase in granulysin expression in the whole population was significant over time. In contrast, the percentage of granulysin-expressing cells was rising in both the CD4^+ ^and the CD8^+ ^populations. On mRNA levels after bacterial or cytokine stimulation all transcripts except NKG5 were upregulated between day 0 and 6 after which a plateau was reached (data not shown). This would mean that granulysin availability is delayed after T-cell activation, but supplies are replenished during Ag-specific responses.

Our results confirmed a rather slow kinetic of granulysin production similar to those described for other proteins associated with T cell terminal differentiation such as granzyme and perforin [41,42]. Studying the kinetics of protein processing proved that IL-2 stimulation leads to processing of granulysin to an active 9 kDa form already in the first week, which is in correlation with data shown by Zheng et al. 2007 where granulysin was detected by day 5, whereas processing under bacterial stimulation occures during the second week of stimulation.

## Conclusion

This first comprehensive study reveals various levels of granulysin gene regulation in human primary lymphocytes under different stimuli. It shows a complex transcriptional expression pattern encoding different mRNA variants through use of alternative splicing, followed by cell type specific translational regulation and slow posttranslational processing.

## Methods

### Generation of LAK cells and dendritic cells (DC)

Human DC and LAK cells were generated *in vitro *from blood-derived precursors as already described [43]. Briefly, human peripheral blood monocytes (PBMC) obtained from venous blood of healthy volunteers were isolated by Ficoll-Paque (Pharmacia Biotech, Uppsala, Sweden) density centrifugation. The PBMC were cultured in RPMI 1640 supplemented with penicillin/streptomycin and 10% human serum (HS) for 2 hours. To obtain dendritic cells (DC), the adherent cells were cultured for 6 days in RPMI 1640 supplemented with penicillin/streptomycin, 5% HS (DC culture medium), recombinant human granulocyte monocyte colony-stimulating factor (rhGM-CSF, 50 ng/ml, Novartis, Basel, Switzerland) and recombinant human IL-4 (100 U/ml, R&D Systems, Abingdon, UK).

The non-adherent cells were cultured in RPMI 1640 supplemented with penicillin/streptomycin (Life Technologies, Paisley, UK), 5% heat-inactivated pooled human A serum (HS, Blood Bank SRK Zürich, Switzerland), and IL-2 (66 U/ml, R&D Systems, Abingdon, UK). On days 0, 4 and 15, phenotypic characterization of the LAK cells was performed by flow cytometry using anti-human CD Abs (see below). On days 4 and 15, different T-cell subpopulations were positively selected by anti-CD4, anti-CD8 and anti-CD56 MicroBeads using Octomacs (Miltenyi Biotec, Bergisch-Gladbach, Germany). Cell purity was more than 90% as assessed by flow cytometry.

### Generation of antigen specific T-lymphocytes, autologous mixed leukocyte-DC culture (MLC)

DC were *Listeria *challenged as described previously [16,16,16,44,43,42,41,41] Briefly, 6 days matured DC cells were transferred to 6-well plates and challenged with opsonized *L. innocua *or *L. monocytogenes *at a multiplicity of infection (MOI) of 10 or Ovalbumin 5 μg/ml for 1 hour at 37°C. Non-internalized bacteria and OVA were removed by washing with PBS before adding media containing 20 μg/ml gentamycin (Sigma, St. Louis, MO). DC washed with equivalent amount of PBS served as a negative control (MOCK). After 16 hours, freshly isolated autologous, nonadherent PBMC in RPMI with 5% HS were added to the challenged DC at the PBMC/DC ratio of 10/1. The MLC was cultured for 6 days before restimulation with fresh *Listeria *challenged autologous DC. At days 0, 6 and 12 flow cytometry phenotypic analysis of the antigen specific T-cells was performed using anti-human CD Abs (see below). Additionally, at day 6 and day 12, CD4^+ ^and CD8^+ ^cells were separated using MACS beads.

### Flow cytometry

For the phenotyping of the PBMC the following Abs were used according the manufacturers instructions: FITC-labeled anti-human CD4, PE-coupled anti-human CD8, FITC-labeled anti-human CD3, PE-coupled anti-human CD56, (all Diatec.Com AS, Oslo, Norway). Cells were analyzed in FC500 flow cytometer (Beckman Coulter, Fullerton, CA) using CXP software (Beckman Coulter).

### Confocal laser scanning microscopy

T-cells or LAK cells were fixed with 1.5% formaldehyde and 1% sucrose in PBS and were cytospun onto glass slides and permeabilized with 0.1% Triton X-100 (Sigma) in PBS for 40 seconds at room temperature. Unspecific binding was blocked with 0.5% BSA (bovine serum albumin, Fluka, Buchs, Switzerland) in PBS for 30 minutes at room temperature. The samples were labeled with guinea pig anti-granulysin antiserum generated in our laboratory as described by Walch et al. [11]. Omitting the first Abs served as control for specificity. For detection FITC-conjugated donkey anti-guinea pig Ab (1/200, Jackson ImmunoResearch, West Grove, PA) was used. For granulysin detection in different LAK subpopulations, the cells were first labeled with anti human CD Abs (see above) for 20 minutes on ice followed by washing in PBS and fixation. Fluorescent labeled specimens were examined using a confocal laser scanning microscope (CLSM SP1, Leica, Heidelberg, Germany).

### Western Blots

LAK-cells or antigen specific T-cells (1–3 × 10^6 ^cells) were lysed in 0.5% Triton X-100, incubated with 0.4 μg/ml DNAse (Sigma-Aldrich) and subsequently mixed with SDS-PAGE sample buffer followed by separation on 15% SDS-PAGE at 200 V for 45 minutes. The proteins were transferred to an Immobilon-P polyvinylidene fluoride transfer membrane (Millipore, Billerica, MA) at 100 V for 75 minutes. The membrane was blocked using 1% casein (Roche, Basel, Switzerland) in TBS-Tween (0.1%) (Sigma-Aldrich) for 30 minutes. Guinea pig anti-granulysin (1:1000) and goat anti guinea pig HRP (1:5000, Sigma-Aldrich) in blocking solution were used for detection. The signal was visualized using ECL plus western blotting detection system (Amersham Biosciences, Uppsala, Sweden).

### RT-PCR

Total RNA was isolated from LAK- or antigen specific T-cells by the RNeasy micro kit from Qiagen (Hilden, Germany) according to the manufacturers instructions. First strand cDNA synthesis was performed using Superscript II reverse transcriptase and oligo (dT) primers (Invitrogen, Carlsbad, CA). PCR was performed using Tag DNA polymerase (Q-Biogene, Irvine, CA) and primers shown in Table 1. As templates for primer design the different transcripts found in GenBank or published by Manning et al. [8] were used. PCR was carried out with RoboCycler (Stratagene, La Jolla, CA) using the following program: initial denaturation for 4 minutes at 94°C, followed by 30 cycles of denaturation at 94°C for 45 seconds, annealing at 58°C for 45 seconds, and extension at 72°C for 45 seconds. Final extension was obtained at 72°C for 8 minutes. RT-PCR products were subjected to electrophoresis on a 1.5–2% agarose gel containing ethidium bromide and were visualized under UV light. For semi quantitative analysis the fluorescent intensity of each band was measured using ImageJ software [44] and normalized relative to the intensity of the actin band from the same cDNA.

**Table 1:** Primers used in RT-PCR

Primer No.	Accesion number	Name of primer	Sequence
1	NM 006433	NKG5 sense A	5'-GTA TCT GTG GTA AAC CCA-3'
2	NM 006433	NKG5 sense B	5'-GCT CCC TGC CCA TAA AAC AG-3'
3	NM 006433	NKG5 sense C	5'-CAT CTC AGC GGC TGC CC-3'
4	NM 006433	exon 1 end sense	5'-GCT CCT TGC AGC CAT GCT C-3'
5	NM 012483	519 sense	5'-GCC TCA TCG GTG GAT CTG CGT-3'
6	NM 012483	ST antisense (ex2)	5'-GCA GGA AGT CTG CCT TGA AC-3'
7	NM 006433	granulysin antisense	5'-GTG CTC GAG TTA CCT GAG GTC CTC ACA GAT CTG-3'
8	NM 001101	actin sense	5'-TAT ACC ATG GGC CGG GAC CTG ACT GAC TAC-3'
9	NM 001101	actin antisense	5'-GTG CTC GAG GAA CCG CTC ATT GCC AAT GGT G-3'

## Authors' contributions

SLG participated in the conception of the study, participated in the performance of the experiments, analyzed the data, and drafted the manuscript. MW designed the experiments, designed the primers, did all revision experiments and gave important intellectual support in data interpretation. HB and CD participated in the analysis and interpretation of the data.

PG provided critical intellectual input to the study and organized financial support. UZ participated in the conception and coordination of the study, contributed to the interpretation of the data and helped to draft the manuscript. All authors read and approved the final manuscript.
